# Influence of Home and School Environments on Specific Dietary Behaviors Among Postpartum, High-Risk Teens, 27 States, 2007–2009

**DOI:** 10.5888/pcd12.140437

**Published:** 2015-05-07

**Authors:** Megan A. Clarke, Debra L. Haire-Joshu, Cynthia D. Schwarz, Rachel G. Tabak, Corinne E. Joshu

**Affiliations:** Author Affiliations: Debra L. Haire-Joshu, Cynthia D. Schwarz, Rachel G. Tabak, Washington University in St Louis, St Louis, Missouri; Corinne E. Joshu, Johns Hopkins Bloomberg School of Public Health, Baltimore, Maryland.

## Abstract

**Introduction:**

The objective of this study was to determine whether perceptions of the home and school food environments are related to food and beverage intakes of postpartum teens.

**Methods:**

Our study was a baseline, cross-sectional analysis of 853 postpartum teens enrolled in a weight-loss intervention study across 27 states from 2007 through 2009. Eight-item scales assessed perceived accessibility and availability of foods and beverages in school and home environments. Associations between environments and intakes were assessed by using χ^2^ and using logistic regression with generalized estimating equations (GEE), respectively.

**Results:**

Overall, 52% of teens perceived their school food environment as positive, and 68% of teens perceived their home food environment as positive. A positive school environment was independently associated with fruit consumption and 100% fruit juice consumption. A positive home environment was independently associated with fruit, vegetable, and water consumption and infrequent consumption of soda and chips (χ^2^ *P* < .05). Having only a positive school environment was associated with fruit consumption (GEE odds ratio [OR], 3.1; 95% confidence interval [CI], 1.5–6.5), and having only a positive home environment was associated with fruit (GEE OR, 2.9; 95% CI, 1.6–5.6), vegetable (GEE OR, 3.1; 95% CI, 1.5–6.2), and water (GEE OR, 2.6; 95% CI, 1.7–4.0) consumption and infrequent consumption of soda (GEE OR, 0.5; 95% CI, 0.3–0.7). Results for positive home and school environments were similar to those for positive home only.

**Conclusion:**

Home and school environments are related to dietary behaviors among postpartum teens, with a positive home environment more strongly associated with healthful behaviors.

## Introduction

Nearly one-third of adolescents are overweight or obese and thus are at greater risk for obesity and its long-term health consequences, such as diabetes, in adulthood (1,2). This risk is significantly heightened for postpartum, teenaged mothers who have sociodemographic and behavioral risk factors for overweight and obesity, such as low socioeconomic status and poor diet (3). Both the school and home environments influence dietary behaviors of teenagers, particularly in low-income and racial/ethnic minority populations (4,5). Aspects of food environments that may be particularly important include availability and accessibility of healthful foods such as fruits and vegetables, low-fat snacks, and low-calorie beverages (4,6–8).

More recent evidence suggests that school-based interventions and policies may not be sufficient to overcome risks posed in other settings (9,10). Reports from the Institute of Medicine suggest that although the school environment is a key target for obesity prevention programs, emphasis is also needed on the role of parents or caregivers in shaping dietary behaviors in the home (7,11).

Little is known about how postpartum teens perceive their food environments and whether those perceptions are related to their dietary behaviors (4,12). In previous work with high-risk, postpartum teens, we found a stronger relationship between the perceived home food environment (vs school) and healthful dietary behaviors (Tabak R, Joshu C, Clarke M, Schwarz C, Haire-Joshu D, unpublished data). Here we aim to build on these findings by examining the associations between perceived school and home food environments and consumption of specific food and beverage items and examining whether relationships vary by body mass index (BMI) and participation in nutrition assistance programs. We hypothesize that positive perceptions of food environments will be associated with healthful food and beverage intakes, and that these associations will differ by type of environment.

## Methods

### Study population

This cross-sectional study includes baseline data from postpartum teens enrolled in the Moms for a Healthy Balance Weight-loss Intervention Study (BALANCE), a group-randomized, nested cohort study with an intervention component designed to reduce postpartum weight retention in young mothers (13). BALANCE was developed in partnership with Parents as Teachers (PAT), a child development–parent education program supported by federal and state funds and delivered free of charge to over 200,000 families in all 50 states (14). For this study, we selected 27 states on the basis of the number of adolescent parents expected in the state.

Detailed methods on the BALANCE intervention have been described elsewhere (13). Briefly, trained PAT parent educators delivered an evidence-based curriculum via home visits, group activities, and online resources. Adolescents were eligible to participate if they were enrolled in the PAT Teen Program, were less than 1 year postpartum, and were not pregnant or planning to become pregnant. We enrolled 1,325 eligible adolescent mothers from 2007 through 2009, and the study concluded in 2010. A total of 141 of the 1,325 teen participants randomized did not complete the baseline assessment, and 45 were missing baseline data for the calculation of BMI, leaving a total of 1,139 with complete data. For this analysis, teens who were underweight at baseline (n = 19) as well as those who reported they were not currently in school (n = 221) were excluded. An additional 46 teen participants did not have information on food environments, leaving a total of 853 included in this analysis. The institutional review board of Saint Louis University and Washington University in St Louis approved this study, and informed consent was obtained from all participants.

### Measures

Teen mothers self-reported characteristics including age, race/ethnicity, current education level, length of time since giving birth (postpartum status), breastfeeding status at baseline, and participation in the Supplemental Nutrition Assistance Program (SNAP) and the National School Lunch Program (NSLP).

Trained staff measured height and weight at baseline in accordance with the National Health and Nutrition Examination Survey (NHANES) study procedures (15). Weight, height, and age data were used to calculate age-appropriate BMI categories, following the Centers for Disease Control and Prevention algorithm (16). BMI was dichotomized as normal (<85th percentile) and overweight/obese (≥85th percentile).

Questionnaire items measuring perceived access of 4 healthful items (fruits and vegetables, low-fat products, low-calorie beverages, and low-calorie snacks) were used to characterize the home and school food environments (17,18). For each environment, 8 statements assessed the availability and selection of healthful items at home (eg, “it is easy to find/there is a large selection of low-fat products in my home”) and ease of purchase and selection of healthful items at school (eg, “it is easy to purchase/there is a large selection of low-fat products in school”). Ratings were scored on a 5-point Likert scale (1 = “strongly agree” to 5 = “strongly disagree”). A mean score of the 8 items was created for the school and home food environments (Cronbach’s α = 0.897 and 0.902, respectively) and dichotomized as less than 3.0 being a positive environment and 3.0 or higher being a negative environment.

Dietary behaviors were assessed using the Snack and Beverage Food Frequency Questionnaire (SBFFQ) developed from our previous work (19,20). A validation study and pilot testing were completed with 60 teen participants. The SBFFQ examined the young mothers’ intake of 31 items during the prior 7 days by asking on how many days, how many times per week, and how much of the item she consumed. Items that were consumed by less than 25% of teens were excluded. Because of the nature and distribution of the data, data on the frequency of specific food and beverage items were collapsed into binary categories of infrequent (0–3 d/wk) and frequent (4–7 d/wk) consumption as a more conservative approach (21).

### Statistical analyses

Descriptive statistics were calculated to evaluate baseline characteristics of all postpartum teens and by positive and negative school and home food environments. Differences in baseline characteristics by environment were assessed by using Pearson χ^2^ tests and *t* tests. Relationships between environments and frequency of food and beverage consumption were assessed by using Pearson χ^2^ tests. To evaluate the relative strength of association between home and school environments and dietary behaviors, we created the following categories: “negative school and home,” “positive school only,” “positive home only,” and “positive school and home.” We used multiple logistic regression with generalized estimating equations (GEE) to account for clustering within a state. Potential confounders including NSLP and SNAP participation, race/ethnicity, age, and postpartum status, were identified on the basis of a priori knowledge and assessed by using a backward selection procedure. Final regression models were adjusted for race/ethnicity, age, and postpartum status, and results were calculated as GEE odds ratios (ORs) and 95% confidence intervals (CIs). To determine whether there were any differences by baseline weight status or participation in nutrition assistance programs, all models were stratified by BMI (ie, normal weight vs overweight/obese) and NSLP or SNAP participation. Data were analyzed by using Stata (Stata Intercooled, version 13; Stata Corp LP).

## Results

The mean age of the postpartum teens was 17 years (range, 12–20) and there were no significant age differences by perceived school or home environment (Table 1). Most teens identified as white (44%), black (29%), or Hispanic (20%). Racial distribution varied significantly by home environment, with a greater proportion of white teens reporting a positive home environment (χ^2^ *P* < .05). Slightly more than half of the teens had a normal BMI, and no significant differences were observed between home or school environment and BMI. Participation in SNAP and NSLP was common (30% and 40%, respectively) and varied significantly by home environment, with a greater proportion of postpartum teens reporting a negative home environment also reporting receiving SNAP and/or NSLP benefits (χ^2^ *P* < .05). Most teens were from neighborhoods in rural or suburban settings, and neighborhood location varied significantly by school environment; teens living in a suburban neighborhood were more likely to perceive a negative school environment (χ^2^ *P* < .05). Approximately 75% of teens were 3 months or more postpartum and 12% reported that they were currently breastfeeding.

**Table 1:** Characteristics of 853 Postpartum Teens and Their School and Home Food Environments,^a^ 27 States, 2007–2009

Characteristic	Total^b^	School		Home	
		Positive	Negative	Positive	Negative
**Total N (%)**	853 (100.0)	442 (51.8)	411 (48.2)	577 (67.6)	276 (32.4)
**Age, y, mean (SD)**	17.4 (1.1)	17.3 (1.1)	17.4 (1.0)	17.4 (1.0)	17.4 (1.1)
**Race/ethnicity, n (%)^c^**					
White	379 (44.4)	193 (43.7)	186 (45.3)	264 (45.7)	115 (41.7)
Black	247 (29.0)	131 (29.6)	116 (28.2)	151 (26.2)	96 (34.8)
Hispanic	173 (20.3)	86 (19.5)	87 (21.2)	121 (21.0)	52 (18.8)
Other/missing	54 (6.3)	32 (7.2)	22 (5.3)	41 (7.1)	13 (4.7)
**BMI^d^, n (%)**					
Normal	480 (56.3)	248 (56.1)	232 (56.4)	314 (54.4)	166 (60.1)
Overweight/obese	373 (43.7)	194 (43.9)	179 (43.6)	263 (45.6)	110 (39.9)
**Education, n (%)**					
9th grade	88 (10.6)	53 (12.4)	35 (8.7)	55 (9.8)	33 (12.3)
10th grade	148 (17.9)	80 (18.7)	68 (17.0)	100 (17.9)	48 (17.9)
11th grade	251 (30.3)	125 (29.2)	126 (31.5)	172 (30.7)	79 (29.5)
12th grade	341 (41.2)	170 (39.7)	171 (42.8)	233 (41.6)	108 (40.3)
**SNAP benefits^c^, n (%)**	254 (30.0)	133 (30.3)	121 (29.6)	155 (27.1)	99 (36.0)
**NSLP benefits^c^, n (%)**	346 (40.8)	188 (42.8)	158 (38.6)	214 (37.4)	132 (48.0)
**Neighborhood^e^, n (%)**					
Rural	345 (40.4)	186 (46.2)	159 (41.7)	237 (44.8)	108 (42.4)
Suburban	260 (33.2)	116 (28.8)	144 (37.8)	176 (33.3)	84 (32.9)
Urban	179 (22.8)	101 (25.1)	78 (20.5)	116 (21.9)	63 (24.7)
**Time since giving birth, n (%)**					
<3 months	158 (25.1)	81 (25.6)	77 (24.5)	116 (27.0)	42 (20.9)
3–6 months	193 (30.6)	107 (33.9)	86 (27.4)	130 (30.3)	63 (31.3)
>6 months	279 (44.3)	128 (40.5)	151 (48.1)	183 (42.7)	96 (47.8)
**Breastfeeding^c^, n (%)**	96 (11.7)	56 (13.2)	40 (10.1)	81 (14.6)	15 (5.6)

Abbreviations: BMI, body mass index; NSLP, National School Lunch Program; SNAP, Supplemental Nutrition Assistance Program.

a See the Methods section for a description of how positive and negative perceptions were determined.

b Counts may not sum to overall total because of missing data.

c Significantly different for home environment, χ^2^ *P* < .05.

d Weight, height, and age data were used to calculate age-appropriate BMI categories, following the Centers for Disease Control and Prevention algorithm (16).

e Significantly different for school environment, χ^2^ *P* < .05.

Overall, the item most likely to be consumed more than 3 days per week was chips, followed by cereal (Table 2). A positive school environment was significantly associated with eating fruit more than 3 days per week, while a positive home environment was significantly associated with eating cereal, fruit, and vegetables on more than 3 days per week and chips and chocolate on 0 to 3 days per week (χ^2^ *P* < .05). When we stratified by baseline BMI, the relationships between a positive home environment and frequency of chips and chocolate consumption were significant only among normal-weight teens (χ^2^ *P* < .05). When we stratified by NSLP and SNAP participation, patterns of frequency of intake of food items were similar to the patterns observed for all teens except 1) the relationship between positive school environment and frequency of fruit consumption was significant only for teens participating in NSLP (χ^2^ *P* < .01), and 2) the relationship between a positive home environment and frequency of fruit consumption was significant only among teens not receiving SNAP benefits (χ^2^ *P* < .01).

**Table 2:** Association Between Frequency of Food and Beverage Items Consumed and School and Home Food Environments^a^ for 853 Postpartum Teens, 27 States, 2007–2009

Item Consumed	Total, N (%)	School		Home	
		Positive, n (%)	Negative, n (%)	Positive, n (%)	Negative, n (%)
**Chips^b^**					
0–3 d/wk	624 (73.2)	319 (72.2)	305 (74.2)	434 (75.2)	190 (68.8)
4–7 d/wk	229 (26.8)	123 (27.8)	106 (25.8)	143 (24.8)	86 (31.2)
**Crackers**					
0–3 d/wk	802 (94.0)	410 (92.8)	392 (95.4)	538 (93.2)	264 (95.7)
4–7 d/wk	51 (6.0)	32 (7.2)	19 (4.6)	39 (6.8)	12 (4.3)
**Granola bars**					
0–3 d/wk	812 (95.2)	417 (94.3)	395 (96.1)	545 (94.5)	267 (96.7)
4–7 d/wk	41 (4.8)	25 (5.7)	16 (3.9)	32 (5.5)	9 (3.3)
**Cakes**					
0–3 d/wk	764 (89.6)	394 (89.1)	370 (90.0)	522 (90.5)	242 (87.7)
4–7 d/wk	89 (10.4)	48 (10.9)	41 (10.0)	55 (9.5)	34 (12.3)
**Cookies**					
0–3 d/wk	785 (92.0)	402 (91.0)	383 (93.2)	531 (92.0)	254 (92.0)
4–7 d/wk	68 (8.0)	40 (9.0)	28 (6.8)	46 (8.0)	22 (8.0)
**Chocolate^b^**					
0–3 d/wk	750 (87.9)	389 (88.0)	361 (87.8)	520 (90.1)	230 (83.3)
4–7 d/wk	103 (12.1)	53 (12.0)	50 (12.2)	57 (9.9)	46 (16.7)
**Hard candy**					
0–3 d/wk	794 (93.1)	412 (93.2)	382 (92.9)	542 (93.9)	252 (91.3)
4–7 d/wk	59 (6.9)	30 (6.8)	29 (7.1)	35 (6.1)	24 (8.7)
**French fries**					
0–3 d/wk	738 (86.5)	381 (86.2)	357 (86.9)	505 (87.5)	233 (84.4)
4–7 d/wk	115 (13.5)	61 (13.8)	54 (13.1)	72 (12.5)	43 (15.6)
**Pizza**					
0–3 d/wk	811 (95.1)	415 (93.9)	396 (96.4)	551 (95.5)	260 (94.2)
4–7 d/wk	42 (4.9)	27 (6.1)	15 (3.6)	26 (4.5)	16 (5.8)
**Cereal^b^**					
0–3 d/wk	646 (75.7)	335 (75.8)	311 (75.7)	418 (72.4)	228 (82.6)
4–7 d/wk	207 (24.3)	107 (24.2)	100 (24.3)	159 (27.6)	48 (17.4)
**Fruit^b^^,^ c**					
0–3 d/wk	712 (83.5)	357 (80.8)	355 (86.4)	468 (81.1)	244 (88.4)
4–7 d/wk	141 (16.5)	85 (19.2)	56 (13.6)	109 (18.9)	32 (11.6)
**Vegetables^b^**					
0–3 d/wk	722 (84.6)	367 (83.0)	355 (86.4)	468 (81.1)	254 (92.0)
4–7 d/wk	131 (15.4)	75 (17.0)	56 (13.6)	109 (18.9)	22 (8.0)
**Water^b^**					
0–3 d/wk	251 (29.4)	130 (29.4)	121 (29.4)	144 (25.0)	107 (38.8)
4–7 d/wk	602 (70.6)	312 (70.6)	290 (70.6)	433 (75.0)	169 (61.2)
**Regular soda^b^**					
0–3 d/wk	456 (53.5)	229 (51.8)	227 (55.2)	337 (58.4)	119 (43.1)
4–7 d/wk	397 (46.5)	213 (48.2)	184 (44.8)	240 (41.6)	157 (56.9)
**100% Fruit juice^b^^,^ c**					
0–3 d/wk	597 (70.0)	292 (66.1)	305 (74.2)	381 (66.0)	216 (78.3)
4–7 d/wk	256 (30.0)	150 (33.9)	106 (25.8)	196 (34.0)	60 (21.7)
**Fruit punch^c^**					
0–3 d/wk	712 (83.5)	358 (81.0)	354 (86.1)	477 (82.7)	235 (85.1)
4–7 d/wk	141 (16.5)	84 (19.0)	57 (13.9)	100 (17.3)	41 (14.9)
**Sports drinks^c^**					
0–3 d/wk	787 (92.3)	397 (89.8)	390 (94.9)	530 (91.9)	257 (93.1)
4–7 d/wk	66 (7.7)	45 (10.2)	21 (5.1)	47 (8.1)	19 (6.9)
**Whole or 2% milk^b^**					
0–3 d/wk	472 (55.3)	234 (52.9)	238 (57.9)	304 (52.7)	168 (60.9)
4–7 d/wk	381 (44.7)	208 (47.1)	173 (42.1)	273 (47.3)	108 (39.1)
**Sweet tea**					
0–3 d/wk	711 (83.4)	371 (83.9)	340 (82.7)	483 (83.7)	228 (82.6)
4–7 d/wk	142 (16.6)	71 (16.1)	71 (17.3)	94 (16.3)	48 (17.4)

a See the Methods section for a description of how positive and negative perceptions were determined.

b Significantly different for home environment, χ^2^ *P* < .05.

c Significantly different for school environment, χ^2^ *P* < .05.

Overall, the beverage item most likely to be consumed more than 3 times per week was water, followed by regular soda (Table 2). A positive school environment was significantly associated with frequent consumption of 100% fruit juice as well as 2 types of sugar-sweetened beverages: fruit punch and sports drinks (χ^2^ *P* < .05). A positive home environment was significantly associated with frequent water, 100% fruit juice, and whole or 2% milk consumption, and infrequent regular soda consumption (χ^2^ *P* < .05). We found similar results when we stratified by baseline BMI; however, the significant relationship between a positive home environment and whole or 2% milk consumption was observed only for overweight/obese teens (χ^2^ *P* < .05). When we stratified by NSLP and SNAP participation, patterns of frequency of intake of beverage items were similar to the patterns observed for all teens except that a positive school environment was significantly associated only with drinking 100% fruit juice more than 3 days per week among teens who did not participate in NSLP (χ^2^ *P* < .05).

When compared with teens reporting negative school and home environments, a positive school environment only was significantly associated with increased odds of frequent fruit consumption (GEE OR, 3.1; 95% CI, 1.5–6.5) (Table 3). Compared with teens reporting negative school and home environments, a positive home environment only was significantly associated with frequent consumption of cereal (GEE OR, 2.3; 95% CI, 1.4–3.7), fruit (GEE OR, 2.9; 95% CI, 1.6–5.6), and vegetables (GEE OR, 3.1; 95% CI, 1.5–6.2) and infrequent consumption of chips (GEE OR, 0.5; 95% CI, 0.3–0.8), and a positive home and school environment was associated with increased odds of frequent cereal (GEE OR, 1.7; 95% CI, 1.1–2.8), fruit (GEE OR, 2.9; 95% CI, 1.6–5.4), and vegetable (GEE OR, 3.2; 95% CI, 1.7–6.2) consumption.

**Table 3:** GEE Logistic Regression Analysis^a^ of Food Environments^b^ and Frequency of Food and Beverage Consumption Among 853 Postpartum Teens, 27 States, 2007–2009

Item Consumed	Negative School and Home (n = 179)	GEE OR (95% CI)		
		Positive School Only (n = 97)	Positive Home Only (n = 232)	Positive School and Home (n = 345)
**Food**				
Chips	1 [Reference]	0.8 (0.4–1.3)	0.5 (0.3–0.8)^c^>	0.8 (0.5–1.1)
Crackers	1 [Reference]	1.9 (0.6–6.1)	1.7 (0.6–4.7)	2.3 (0.9–5.9)^d^
Granola bars	1 [Reference]	3.8 (0.9–17.0)^d^	3.5 (0.9–13.6)^d^	3.4 (0.9–12.8)^d^
Cakes	1 [Reference]	1.0 (0.5–2.2)	0.6 (0.3–1.3)	0.8 (0.5–1.5)
Cookies	1 [Reference]	1.3 (0.5–3.1)	0.9 (0.4–1.9)	1.2 (0.6–2.4)
Chocolate	1 [Reference]	1.6 (0.9−3.1)	0.7 (0.4–1.3)	0.6 (0.3–1.0)
Hard candy	1 [Reference]	1.1 (0.5–2.6)	0.7 (0.3–1.5)	0.7 (0.3–1.3)
Fries	1 [Reference]	1.1 (0.5–2.1)	0.7 (0.4–1.3)	0.8 (0.5–1.4)
Pizza	1 [Reference]	1.5 (0.5–4.1)	0.5 (0.2–1.4)	1.2 (0.5–2.5)
Cereal	1 [Reference]	1.2 (0.7–2.4)	2.3 (1.4–3.7)^e^	1.7 (1.1–2.8)^c^
Fruit	1 [Reference]	3.1 (1.5–6.5)^e^	2.9 (1.6–5.6)^e^	2.9 (1.6–5.4)^b^
Vegetables	1 [Reference]	1.3 (0.5–3.3)	3.1 (1.5–6.2)^e^	3.2 (1.7–6.2)^b^
**Beverage**				
Water	1 [Reference]	1.3 (0.8–2.1)	2.6 (1.7–4.0)^e^	1.8 (1.2–2.6)^e^
Regular soda	1 [Reference]	1.4 (0.8–2.3)	0.5 (0.3–0.7)^e^	0.7 (0.5–1.0)^c^
100% Fruit juice	1 [Reference]	1.5 (0.8–2.6)	1.9 (1.2–2.9)^e^	2.3 (1.5–3.6)^e^
Fruit punch	1 [Reference]	1.4 (0.7–2.7)	1.1 (0.6–1.9)	1.5 (0.9–2.6)
Sports drinks	1 [Reference]	2.1 (0.8–5.5)	1.1 (0.4–2.6)	2.0 (1.0–4.4)^d^
Whole or 2% milk	1 [Reference]	1.2 (0.8–2.2)	1.5 (1.0–2.2)^e^	1.6 (1.1–2.3)^e^
Sweet tea	1 [Reference]	0.6 (0.3–1.1)	0.7 (0.4–1.2)	0.8 (0.5–1.3)

Abbreviations: GEE, generalized estimating equations; OR, odds ratio; CI, confidence interval.

a Adjusted for race, age, and length of time since giving birth.

b See the Methods section for a description of how positive and negative perceptions were determined.

c *P* < .01.

d *P* < .1, significant for trend.

e *P* < .05.

Reporting only a positive school environment was not significantly associated with frequent consumption of any beverage items. Compared with teens reporting negative school and home environments, teens reporting a positive home environment only had increased odds of frequent water (GEE OR, 2.6; 95% CI, 1.7–4.0) and 100% fruit juice (GEE OR, 1.9; 95% CI, 1.2–2.9) consumption and infrequent consumption of regular soda (GEE OR, 0.5; 95% CI, 0.3–0.7). Compared with teens reporting negative school and home environments, teens reporting both positive home and school environments had similar results to those reporting only a positive home environment. Teens reporting both a positive home and school environment had significantly greater odds of frequent 100% fruit juice (GEE OR, 2.3; 95% CI, 1.5–3.6) and water consumption (GEE OR, 1.8; 95% CI, 1.2–2.6) and infrequent consumption of regular soda (GEE OR, 0.7; 95% CI, 0.5–1.0) than those reporting both negative home and school environments. Relative relationships between school and home food environments and food and beverage item consumption did not vary by baseline BMI. Significant associations between the positive school food environment and frequent consumption of healthful items such as fruit (GEE OR, 4.8; 95% CI, 1.6–14.6) and 100% fruit juice (GEE OR, 2.4; 95% CI, 1.1–5.6) were observed only among teens participating in the NSLP. The relationships between a positive home environment and both positive home and school environments did not differ substantially by NSLP participation. The relationship between the positive school food environment and dietary intake did not differ by SNAP participation, but significant associations between a positive home environment and infrequent consumption of unhealthful items such as chips (GEE OR, 0.4; 95% CI, 0.2–0.8) and soda (GEE OR, 0.2; 95% CI, 0.1–0.5) were observed only among teens who received SNAP benefits. The same patterns were generally observed for both positive home and school environments.

## Discussion

Our findings indicate that the school and home food environments have differential relationships with food and beverage intakes. Our findings were similar to those from other studies: we found that a perceived positive school environment was primarily related to healthful eating behaviors such as frequent fruit or 100% fruit juice intake but not unhealthful eating behaviors (22,23). In contrast, a perceived positive home environment was associated with frequent consumption of a wider variety of healthful items as well as infrequent consumption of unhealthful food and beverage items such as soda and chips. Our findings regarding the impact of positive school and home food environments suggest that for certain items consumed by teens, the major benefit lies within the home environment. This study contributes to our understanding of the relationship between both the home and school food environment and dietary behaviors of this understudied population of postpartum teens.

Numerous studies have documented the impact of policy and behavioral interventions promoting healthful school food environments on positive dietary change in youth (8,13,24). Increased access to fruit and various juices may be a result of enhanced school wellness and nutrition policies, which promote access to and availability of select foods (13,25). In addition, school meal programs such as NSLP that promote fruit and vegetable intake in school environments provide opportunities for increased fruit and vegetable consumption among low-income teens (26). However, easy access to and availability of high-calorie and high-fat snacks and sugar-sweetened beverages (ie, “competitive foods”) that had been commonly sold in vending machines and at after-school fundraisers may have limited the effectiveness of school food policies and the influence of a positive school environment on teens’ eating behaviors (24,27). Our results as well as findings from other studies indicate that while a positive school environment may be related to frequent intake of certain healthful food and beverage items, it was not associated with infrequent intake of unhealthful items such as sweet and salty snacks and sugar-sweetened beverages (4,22,23,28). These findings support the importance of recent changes in school food policies that limit access to unhealthful snacks by requiring improvements in the nutrition content of vending machine foods.

Unlike childhood obesity interventions in the school setting, interventions conducted in the home have not been common. Many of these interventions have focused on individual behavior change without addressing the home food environment, limiting their impact on dietary intake and other obesity-related outcomes (7,10). Results from our study are consistent with the literature suggesting the home environment has an important relationship with dietary intake among adolescents (9,29). The home food environment represents a substantial part of the full environmental context in which a postpartum teen grows, develops, eats, and behaves and is guided by “family policies” informed by tradition and culture as well as neighborhood and economic environment (7,29). Additionally, new mothers may be particularly aware of and sensitive to the health quality of their home setting (13). Our findings suggest the multiple and variable influences of a positive home environment have the added benefit of reducing unhealthful behaviors among postpartum teens.

To our knowledge, ours is the first study to examine whether associations between the school and home environments and food and beverage intake differ by participation in nutrition assistance programs. Other studies have shown mixed associations between SNAP and NSLP participation and dietary behaviors (9,30). Our findings suggest that the relationship between the food environment and frequency of consumption of certain items may be stronger among postpartum teens receiving nutrition assistance than those who did not receive assistance. Future research is needed to determine whether there are differences in the relationship between the environment and dietary behavior among teens that do and do not participate in nutrition assistance programs.

Our study has several limitations. This was a cross-sectional analysis; thus, we cannot evaluate causal relationships. Furthermore, reliance on self-reported data for dietary intake may be subject to recall bias and measurement error such as underreporting of items consumed. We attempted to limit potential misclassification by collapsing food and beverage frequency into dichotomous categories, but misclassification is a concern when using SBFFQ data (6,20). Although we were not able to compare data on the school and home environments with objective measures, studies have shown that perceived quality of home- and school-based settings independently influences dietary behavior (4,12). Therefore, we consider using perceptions of the school and home food environments a strength of this study, particularly because we are among the first to address perceptions of the school and home food environments and how they are related to behavior. Additional strengths of this study include a large and nationally representative sample of postpartum teens, an understudied population with a high risk for overweight and obesity.

Our study highlights the importance of both the school and home food environments and their differential relationships with dietary behaviors among teens at high risk for obesity. Further work targeting interventions across both home and school environments simultaneously are needed. In addition, it is important to understand whether different subpopulations respond differently to environmental influences to tailor effective obesity interventions and policies. Improving the home environment may be particularly important among this population of teen mothers who directly control the food environment of their infants. Environmental interventions in this high-risk and hard-to-reach population may not only be important for reducing the risk of adult-onset obesity in the teenaged mother but may also have substantial impact in minimizing the intergenerational transfer of obesity-related behaviors to offspring (13).
